# Did Arkansas’ Medicaid Patient-Centered Medical Home Program Have Spillover Effects on Commercially Insured Enrollees?

**DOI:** 10.1177/0046958019900753

**Published:** 2020-01-16

**Authors:** Jesse M. Hinde, Nathan West, Samuel J. Arbes, Marianne Kluckman, Suzanne L. West

**Affiliations:** 1 RTI International, Research Triangle Park, NC, USA

**Keywords:** patient-centered medical home, spillover, all-payer claims data, expenditures, utilization

## Abstract

Patient-centered medical homes are increasingly being implemented by state Medicaid programs to incentivize high-quality, coordinated care and ultimately lower health care spending. This study examined whether the Arkansas Medicaid Patient-Centered Medical Home Program’s practice-wide transformation activities had spillover effects on commercial beneficiaries. We used difference-in-differences to compare utilization and expenditures of commercially insured enrollees as their practices received Medicaid patient-centered medical home certification on a rolling basis between 2014 and 2016. We found a 5.7% increase in outpatient visits and 13% higher expenditures among early adopting practices. Even without associated reductions in costly emergency department visits or inpatient hospital admissions, decisionmakers should not lose sight of the potential value of increased engagement in and coordination of professional services for a population with high unmet health needs. Our results also emphasize that states can leverage Medicaid to spur system-wide transformation, and the investments generate spillover effects beyond those covered directly by Medicaid.

**What do we already know about this topic?**
Patient-centered medical homes are increasingly being implemented by state Medicaid programs to incentivize high-quality, coordinated care and ultimately lower health care spending.
**How does your research contribute to the field?**
This study found that the Arkansas Medicaid Patient-Centered Medical Home Program’s practice-wide transformation activities had spillover effects on commercial beneficiaries at early adopting practices, increasing access to outpatient care.
**What are your research’s implications toward theory, practice, or policy?**
Our results also emphasize that states can leverage Medicaid to spur system-wide transformation, and the investments generate spillover effects beyond those covered directly by Medicaid.

## Background

Arkansas has historically faced many health care challenges and is often at the bottom of state rankings of overall health and health care access.^1,2^ Several recent initiatives have sought to improve the state’s health care system through multipayer system transformation.^3^ Since 2012, Arkansas has initiated several reforms to promote payment models that reward providers who consistently deliver high-quality, coordinated, and guideline-concordant care to patients. These include participation in the Centers for Medicare & Medicaid Services (CMS)-funded multipayer Comprehensive Primary Care (CPC) Initiative, and in 2014, the state’s own designated patient-centered medical home (PCMH) model for use in Medicaid. Using funding from a CMS State Innovation Models (SIM) award, Arkansas adapted PCMH requirements used in CPC to accommodate features of practices that served many Medicaid patients but were not eligible for CPC, such as pediatric practices.^3^ State Innovation Models funds were used to support practice transformation assistance that enabled practices to meet PCMH requirements and state-level PCMH model management, such as data analytics that fed into practice feedback reports.

Arkansas Medicaid pays each participating practice a per-member per-month (PMPM) fee that varies according to the health risk of each Medicaid fee-for-service enrollee to cover care coordination and more intensive case management. Each practice participating in the Medicaid PCMH model receives a risk-adjusted Medicaid PMPM that was $4 on average but reached as high as $30. The low average for PMPM reflected that many traditional Medicaid beneficiaries were children with low acuity but clinics with higher acuity or older populations often received a more substantial Medicaid PMPM. Additionally, some practices meeting minimum Medicaid panel size requirements are eligible for one-sided risk shared savings payments if they meet financial and quality targets.^4^

Although the PMPM was tied specifically to Medicaid beneficiaries, the practice transformation efforts were payer agnostic and practice wide, affecting all patients. Practice-wide transformation activities included 24/7 live voice access to a health professional, care coordination, flexible same-day visit scheduling, use of Meaningful Use–certified electronic health records, and assessment of opportunities for practice improvement.^5^

For practices that became Medicaid PCMHs, enhanced care coordination and access to primary care services were expected to curtail avoidable, higher cost utilization such as emergency department (ED) visits and inpatient admissions and thus control total expenditures. There is not yet a broad consensus in the literature on the effects of PCMHs on service utilization and expenditures. A systematic review and meta-analysis of PCMHs suggests that they are associated with increased access to preventive services and reduced ED visits among older people but that effects on inpatient admissions and costs are inconclusive.^6^ One recent study suggests that PCMHs associated with accountable care organizations (ACO) may yield cost savings,^7^ and another study of state multipayer advanced primary care demonstrations found mixed effects on Medicare enrollee utilization and expenditures.^8^

Because PCMH certification under Arkansas’ Medicaid PCMH Program require its adoption to be practice wide, changes in care coordination, same-day access, and other quality improvements at the practice level would naturally spill over to non-Medicaid patients. An evaluation of the Arkansas Medicaid PCMH Program found increases in the rate of physician visits and decreases in inpatient admissions and expenditures among Medicaid beneficiaries after 1 year, relative to practices that became PCMHs in later years.^9^ Building on findings in the Medicaid population, this study assessed whether the Arkansas Medicaid PCMH Program had spillover effects on the commercially insured populations being served by the participating practices.

Spillover effects to other patient populations are not yet well understood.^7^ One study from New York City suggested that patients outside the Emblem PCMH health plan were unaffected by PCMH implementation; however, there was strong evidence that transformation was not practice wide.^10^ Another study from Illinois’ Medicaid reform suggests that cost savings and utilization changes spilled over to non-PCMH Medicaid enrollees but did not examine spillover on commercial enrollees.^11^ In the literature on ACOs, there is some evidence that Blue Cross Blue Shield of Massachusetts’ commercial ACO model resulted in lower expenditures for Medicare beneficiaries and little evidence that Blue Cross Blue Shield of Massachusetts’ Medicare ACO model spilled over to commercial beneficiaries.^12,13^

## Methods

### Study Design

To assess spillover effects of the Medicaid PCMH model on commercial enrollees, we used a difference-in-differences (DD) quasi-experimental study design with practice fixed effects that exploited the rolling implementation of PCMH adoption across different practices between 2014 and 2016. As shown in Exhibit 1, practices were part of the comparison group until they began receiving PMPM payments, after which they became part of the intervention group. The rolling implementation allowed us to use commercial plan enrollees seen by the later-adopting practices as a comparison group while in the pre-PCMH phase.

**Exhibit 1.** Arkansas Medicaid PCMH Implementation between 2014 and 2016.

Analytic year	Intervention group	Comparison group
2013	*No* practices	*All* practices
2014	Practices that became certified as a PCMH in early and mid-2014	Practices that became certified as a PCMH in 2015 or 2016
2015	Practices that became certified as a PCMH in 2014 or 2015	Practices that became certified as a PCMH practice in 2016
2016	*All* practices	*No* practices

*Source.* Authors’ analysis.

*Note.* There were 111 early 2014 PCMH practices, 13 mid-2014 PCMH practices, 24 2015 PCMH practices, and 27 2016 PCMH practices. PCMH = patient-centered medical home.

Practices were eligible to participate in the PCMH Program if they were a primary care practice, rural health clinic, or area health education center; enrolled in the ConnectCare Primary Care Case Management (PCCM) program; did not participate in the PCCM shared savings pilot; and had at least 300 Medicaid beneficiaries at the time of enrollment.^14^ The Medicaid PCMH Program grew from approximately 120 practices in 2014 to 180 practices in 2016, representing approximately 72% of eligible practices.^5,9^ Participating PCMHs were mostly family care practices, and about a third were pediatric practices.^9^

The natural variation in adoption allowed us to control for secular changes outside of PCMH adoption that occurred in the intervention and comparison groups, specifically, to control for the influence of the 2014 Affordable Care Act (ACA). By observing repeated adoptions of PCMH in 2014, 2015, and 2016, we can detect effects separate from the ACA that are attributable to PCMH. We also estimate a model that compares the early 2014 PCMH adopters with the mid-2014, 2015, and 2016 PCMH adopters, which directly conditions out the influence of the ACA.

We limited the sample to the 175 practices that were eventually certified in the PCMH Program for at least one full calendar year, as reported by the Arkansas Department of Human Services. Given the penetration of the Medicaid PCMH program and PCMH implementation through CPC, there were too few practices left that could serve as comparison practices. Arkansas stakeholders also reported that practices that would not participate in the Medicaid PCMH program or CPC were substantively different in terms of practice size, ownership, and capabilities to transform into an advanced care home.

We kept a practice, along with its attributed enrollees, in the analytic sample during the time the practice maintained its PCMH certification; we excluded the practice from the sample after de-certification. Thirty-one practices let their PCMH certification lag or their PCMH certification was suspended or terminated during the observation window. Two practices in the sample also participated in the CPC initiative.

Because PCMH is a practice-level intervention, we included in our patient analytic sample only commercial enrollees who received most of their care at a Medicaid PCMH practice in a given year. We did not have information on whether people were assigned to a primary care provider (PCP) by their insurer, so we used an algorithm to attribute enrollees to a practice each calendar year. Because the Arkansas All-Payer Claims Database (APCD) was not available before 2013, we are unable to prospectively attribute individuals to a PCP. Individuals were attributed to a practice each year if they had a plurality of their total PCP visits to that PCMH practice and if they had at least 3 PCP visits to the assigned PCMH practice.

Before attribution, there were 1 044 205 unique commercially insured enrollees between 2013 and 2016.^15^ We limited the sample to the 293 583 unique enrollees with at least 6 months of continuous enrollment in a commercial insurance plan or 9 months of noncontinuous enrollment in a commercial insurance plan during the calendar year. Of those, 124 493 enrollees were attributed to a PCMH practice. The final analytic sample included 121 073 unique, unweighted enrollees.^16^

### Data

We used commercial claims data from 2013 to 2016 extracted from the Arkansas APCD, which includes claims from individual market, small employer, large employer, and state/federal health plans. Claims from self-insured employers are largely not included in the Arkansas APCD. The individual market includes those who purchased health care coverage through qualified health plans (QHPs) offered under the Arkansas Health Insurance Marketplace. Low-income adults who received health care coverage under Arkansas’s 2014 Medicaid expansion are also considered commercially insured in this analysis because of the way Arkansas expanded Medicaid. Arkansas used a Medicaid Section 1115 waiver, known as the “Private Option,” to enroll these newly eligible adults in the same QHPs that offered individual policies through the Arkansas Health Insurance Marketplace. Thus, “Marketplace” plans included lives covered under expansion of Medicaid eligibility to the lowest income adults in Arkansas, lives covered with help from premium subsidies offered under the ACA, and people who purchased health coverage on the Marketplace without being eligible for subsidies.

We examined 2 types of outcomes: expenditures and service utilization. Expenditure categories included total, professional, prescription drug, inpatient facility, and outpatient facility. All expenditures were adjusted to 2014 dollars using the Bureau of Labor Statistics Consumer Price Index for medical care. Claims were aggregated to the quarterly level and were divided by 3 to obtain a PMPM interpretation. Service utilization indicators included any inpatient visit, any primary care visit, any specialist visit, and any ED visit. Outcomes were weighted the fraction of the quarter during which the enrollee was eligible for the analysis.

Demographic covariates in the model included age, age squared, sex, and having a behavioral health diagnosis during the quarter. We controlled for behavioral health conditions because prior research suggests that PCMH implementation may affect this population differently.^17^ Quarterly insurance characteristics included having prescription drug coverage, having Marketplace coverage, having a claim flagged as Medicaid Private Option, insurance product type (preferred provider organization, point of service, or other commercial plan), and insurance market type (individual, small employer, large employer, or other). Because practice-level characteristics were unmeasured in this dataset, we used practice-level fixed effects (described further in the “Empirical Approach” section) to account for any potential bias related to a practice’s readiness to take on transformation activities.

We also included county-level characteristics from the 2013 to 2015 Area Health Resources File to control for local socioeconomic conditions and health service area characteristics. Values for 2015 were also used in 2016 because the values in the relevant variables did not change between 2015 and 2016. In the instance of missing county information, we assigned the median value for the state for that year. We included calendar-year, county-level measures of the percentage of the population under the federal poverty level, median age, percentage uninsured, percentage living in a metropolitan area statistical area, and hospital beds per 1000 population.

### Empirical Approach

Our main statistical approach was a 2-way fixed-effects DD, shown in equation 1

(1) $$Y_{ict}=\beta_{0}+\beta_{1}PCMH_{ict}+Q_{t}+P_{c}+\gamma X_{ict}+\varepsilon_{ict},$$

where $Y_{ict}$ was the outcome of interest for individual, *i*, in practice *c*, during quarter *t*, $PCMH_{ict}$ was equal to 0 while the practice was not certified as a PCMH and switched to 1 when the practice was certified, $Q_{t}$ was a fixed effect for the quarter in the study period, $P_{c}$ was a practice fixed effect, X was a vector of patient demographic and county characteristics, and $Q_{t}$ was a random error term.

The practice fixed effects ($P_{c}$) captured differences across practices. Typically, a DD specification would control for baseline differences between enrollees in the attributed intervention and comparison groups. However, because each practice was eventually certified as a PCMH, that term was omitted because of collinearity with the practice fixed effects. The quarterly fixed effects ($\varepsilon_{ict}$) captured the change in the outcome among practices that had not yet achieved PCMH certification. $\beta_{1}$ is the DD effect and shows whether the difference between practices that have and have not yet achieved PCMH certification increased ($\beta_{1}>0$) or decreased ($\beta_{1}<0$) after PCMH certification.

We estimated a variation of the main statistical model that estimates the effect for early 2014 adopters relative to the other 3 later PCMH-adopting groups to (1) directly control for the influence of the ACA and (2) investigate whether there were larger changes for the early 2014 practices. The early 2014 practices, as postulated by the state at the time, were likely more ready for practice transformation and may be more likely to demonstrate changes in the study outcomes.^3^

(2) $$\begin{array}{l} Y_{ict}=\beta_{0}+\beta_{1}PCMH_{ict}+\beta_{2}Early2014_{ict}\\ +\beta_{3}Early2014_{ict}*PCMH_{ict}+Q_{t}+P_{c}+\gamma X_{ict}+\varepsilon_{ict} \end{array}$$

In equation 2 for this second model, we interact a dummy for the early 2014 practices ($Early2014_{ict}$) with the PCMH indicator. The quarterly fixed effects, practice fixed effects, X vector, and error term were the same as in equation 1.

$\beta_{1}$ was the effect of PCMH certification for the non-early 2014 groups and $\beta_{2}$ was the pre-PCMH difference between early 2014 adopters and the other adopting groups in 2013. $\beta_{3}$ captured whether the early 2014 adopters, who had 3 years of post-PCMH observations, saw differential changes in their outcomes associated with PCMH relative to the groups with less exposure to the PCMH Program during 2014 to 2016. Because the later-adopting practices have pre-PCMH observations during the initial implementation of the ACA, $\beta_{3}$ also conditioned out the changes associated with the ACA during that time as long as the early 2014 adopting practices were not affected differently by the ACA than the later-adopting practices.

Expenditure models were estimated using weighted ordinary least squares. For the utilization models, we used a weighted logistic regression model and multiplied the marginal effect from the logistic regression models by 100 or 1000, as appropriate, to obtain approximate rates of utilization per 100 or 1000 enrollees.^18^ All enrollees were weighted by the fraction of days they were eligible under the insurance plan in the quarter. Standard errors were clustered at the PCMH practice level to account for correlation in the error term between multiple enrollees in practices.

DD is subject to the parallel trends assumption that there are similar pre-PCMH trends between the intervention and comparison groups. Due to the limitations of the Arkansas APCD not being available before 2013, we were limited in testing the parallel trends assumption empirically. First, we estimated a linear model that interacted the PCMH indicator with a linear time trend and no significant differences were found across all study outcomes. Second, using the 4 quarters of data in the common baseline year 2013, we interacted a linear time trend with an indicator for each cohort with the 2016 practices as the referent group. Presented in *Appendix Table A1*, we do find statistically significant differences in inpatient expenditures, inpatient visits, and total expenditures between the 2015 and 2016 PCMH groups.^18^ Appendix Figures A1 to A9 present unadjusted trends across time and do not show strong evidence of differential trends in the pre-PCMH period other than inpatient expenditures. With short pre-PCMH period as a noted limitation and differences in inpatient expenditure, we present evidence that the parallel trends assumption may be satisfied.

## Results

### Descriptive Results

Exhibit 2 presents unadjusted, annualized outcomes and characteristics of the analytic sample by (1) PCMH status (pre- and post-adoption of PCMH), (2) calendar year, and (3) baseline year (2013) observations by PCMH group. With these different perspectives, we examined unadjusted pre- and post differences by PCMH status, the secular change across time, and baseline differences among the PCMH adopter groups, respectively.

**Exhibit 2.** Weighted Annual Sample Characteristics, 2013 to 2016.

Sample characteristic	Annualized by PCMH status		Annualized by calendar year				Annualized by PCMH group in the baseline period (2013)			
	Pre-PCMH (1)	Post-PCMH (2)	2013 (3)	2014 (4)	2015 (5)	2016 (6)	Early 2014 PCMH adopters (7)	Mid-2014 PCMH adopters (8)	2015 PCMH adopters (9)	2016 PCMH adopters (10)
Total expenditures	$4044	$5784	$3427	$4963	$6036	$6555	$3243	$4402	$4304	$3432
Professional expenditures	$1561	$2046	$1400	$1921	$2125	$2206	$1348	$1661	$1600	$1431
Prescription expenditures	$870	$1270	$835	$1007	$1307	$1466	$809	$963	$1063	$797
Inpatient facility expenditures	$693	$1077	$459	$873	$1156	$1279	$405	$721	$813	$445
Other facility expenditures	$920	$1390	$734	$1162	$1448	$1604	$681	$1057	$828	$759
Any inpatient admission (%)	4.4	7.1	3.1	5.8	7.8	8.1	2.8	4.4	4.4	3.2
Any specialist visit (%)	42.5	48.6	43.0	46.3	49.3	50.7	42.6	50.3	41.3	39.9
Any ED visit (%)	24.1	31.5	20.7	29.6	33.0	33.5	20.5	20.7	22.8	21.7
Age	31.7	34.5	28.0	33.0	35.1	35.9	26.5	33.5	36.1	29.9
Female (%)	58.3	61.0	55.2	61.0	61.1	61.4	54.7	57.4	59.9	54.6
BH diagnosis (%)	20.0	23.9	18.5	22.6	24.6	25.4	18.2	17.6	23.2	19.6
Lives in MSA (%)	53.0	52.2	57.2	51.7	51.8	50.0	59.4	58.1	36.0	49.1
Has prescription drug coverage (%)	87.2	90.1	83.9	89.6	90.0	91.1	83.5	85.0	90.7	82.8
Marketplace plan (%)	20.8	43.0	0.0	36.3	46.2	48.2	0.0	0.0	0.0	0.0
Insurance product type										
Other commercial insurance (%)	15.5	19.5	14.2	16.8	19.3	22.0	13.2	14.9	16.1	19.4
Insurance type—PPO (%)	54.9	59.3	49.8	61.4	60.1	57.5	52.6	41.9	42.2	40.6
Insurance type—PoS (%)	21.8	14.3	27.2	16.0	13.9	12.9	25.9	32.4	30.1	30.2
Insurance market type										
Individual market plan (%)	43.7	60.5	27.8	54.5	63.3	64.7	26.9	33.9	30.6	27.1
Large employer plan (%)	33.3	24.4	42.7	26.6	23.2	22.4	43.5	40.0	41.6	40.2
Small employer plan (%)	9.2	6.7	11.8	7.4	6.4	5.9	11.9	11.8	12.8	11.2
Unweighted *N*	52 970	134 256	30 989	46 121	51 755	52 499	23 209	3181	1315	3284
Weighted *N*	55 881	140 627	32 107	48 846	54 078	55 027	24 040	3287	1371	3408

*Source.* Arkansas All-Payer Claims Database, 2013 to 2016.

*Note.* Numbers in brackets refer to the column numbers. Column numbers are referenced in the text discussing this exhibit. BH = behavioral health; ED = emergency department; MSA = metropolitan statistical area; PCMH = patient-centered medical home; PoS = point of service; PPO = preferred provider organization.

We found that utilization and expenditures increased substantially after PCMH implementation (columns 1 and 2) and over time (columns 3-6). After PCMH implementation, average annual total expenditures increased by 43%, from $4044 to $5784. The percentage of enrollees with an inpatient admission in a given year increased from 4.4% to 7.1%, an increase of 61%, and average inpatient facility expenditures increased by 55%. The other expenditure and utilization outcomes increased between 31% and 46%.

As shown in column 1 of *Exhibit 2*, before practices became a certified PCMH, the average enrollee was in his or her early 30s, and 58% were female. Most of the sample lived in a metropolitan statistical area, except among the 2015 PCMH practices, which were more rural. Approximately 20% to 25% of the sample had a diagnosed behavioral health condition. More than 80% of the sample had pharmacy benefits, most were enrolled in a preferred provider organization, and most were enrolled in an individual market plan.

Expenditures per enrollee and rates of utilization increased in each year after 2013, as shown by columns 3 to 6. By 2016, the average value for each expenditure and utilization measure nearly doubled relative to 2013. This trend was driven by the influx of new enrollees through the Health Insurance Marketplace that was introduced in 2014, increasing the sample size by roughly 50%. Approximately 36% of people in 2014 had Marketplace coverage, and this percentage grew to nearly 50% by 2015. *Appendix Table A2* provides summary statistics for the Marketplace and non-Marketplace populations and shows that the Marketplace population, which includes Private Option and Medicaid expansion enrollees, had much higher expenditures and used more services.^19^ Many people in the Marketplace may have previously not had insurance coverage and may have newly gained access to care. Therefore, the increased rates of utilization likely reflect pent-up demand for services among these newly enrolled individuals. Because of these differences, we also estimated separate models that exclude the Marketplace sample.

In terms of baseline differences in 2013, as displayed in columns 7 to 10 of *Exhibit 2*, the mid-2014 and 2015 PCMH groups appear to have higher expenditure patterns at baseline than the early 2014 and 2016 groups, although they had similar service use except for specialist visits. The demographics of the mid-2014 and 2015 PCMH practices were older and more rural, suggesting that their higher expenditure patterns could be associated with costlier health care services.

### Regression Results

*Exhibit 3* presents estimates of the spillover effects of the Medicaid PCMH on commercial enrollees using DD. The first model tests spillover effects for all PCMH practices, and the second model tests the difference in effects between the early 2014 adopters and all other PCMH practices. For each outcome, we showed the 2013 adjusted mean, the DD estimate, and the relative difference that compares the DD estimate with the 2013 adjusted mean.

**Exhibit 3.** Estimated Spillover Effects of Medicaid PCMH Adoption on PMPM Expenditures and Quarterly Rates of Utilization, 2013 to 2016.

Study outcome	Weighted N	Model 1: Spillover effects across all PCMH practices			Model 2: Spillover effects for early 2014 PCMH practices		
		2013 adjusted mean	DD estimate (SE)	Relative difference (%)	2013 adjusted mean, CG practice	DD estimate (SE)	Relative difference (%)
Expenditures							
Total expenditures	704 419	292.8	−0.04 (13.5)	−0.01	275.4	35.7* (20.8)	13.0
Professional expenditures	704 419	119.4	−2.3 (3.7)	−1.9	114.9	20.2*** (5.3)	17.6
Pharmaceutical expenditures	704 419	71.1	5.4 (4.08)	7.6	68.9	−1.7 (7.28)	−2.5
Inpatient facility expenditures	704 419	39.9	−5.6 (7.7)	−13.9	33.5	14.5 (13.8)	43.3
Outpatient facility expenditures	704 419	62.4	2.4 (6.1)	3.8	58.1	2.7 (7.3)	4.7
Service utilization							
Inpatient admissions per 1000 enrollee quarters	701 897	9.1	0.26 (0.72)	2.9	8.2	1.0 (1.5)	11.8
Primary care visits per 100 enrollee quarters	704 416	70.5	0.32 (0.39)	0.5	70.1	1.0** (0.45)	1.5
Specialist visits per 100 enrollee quarters	704 384	18.6	−0.18 (0.4)	−2.0	18.3	1.1** (0.5)	5.7
Emergency department visits per 1000 enrollee quarters	704 373	67.0	3.2 (1.9)	4.7	65.4	5.0 (4.2)	7.6

*Source.* Arkansas All-Payer Claims Database, 2013 to 2016.

*Note.* DD models were estimated using ordinary least squares for expenditure outcomes and maximum likelihood logits for service utilization outcomes. The relative difference is the DD estimate expressed as a percentage of the 2013 adjusted mean. DD = difference-in-differences; PCMH = patient-centered medical home; PMPM = per member per month; SE = standard error; CG = comparison group.

* *P* < .1. **P < .05. ***P < .01.

In Model 1, we did not find robust evidence of effects of Medicaid PCMH PMPM payments on commercial enrollees. PCMH implementation was associated with a statistically insignificant decrease of $0.04 in average monthly total expenditures, relative to the pre-PCMH period and comparison group. Compared with the adjusted average monthly total expenditures in 2013 ($292.80), this estimate implies a relative decrease of 0.01%. The effects of the full Arkansas Medicaid PCMH Program on the remaining expenditure and service utilization outcomes were also not significant.

For Model 2, we found that early PCMH certification was associated with a relative increase in total and professional expenditures and PCP and specialist visits. Practices certified in 2014 had a $35.74 greater increase in total expenditures relative to the PCMH practices that were certified later in the program, representing a relative increase of 13.0% (*P* < .1). Early PCMH certification was also associated with a statistically significant $20.21 increase in average monthly professional expenditures, a 17.6% relative increase (*P* < .01). The increase in total expenditures and professional expenditures corresponded to a 5.7% relative increase in specialist visits per 100 enrollee quarters and a 1.5% increase in primary care visits per 100 enrollee quarters.

### Sensitivity Analyses

We conducted several sensitivity analyses to test the robustness of the results against different assumptions. First, we tested the influence of the ACA by re-estimating the models using only the non-Marketplace enrollees. We still found no statistically significant effects on the study outcomes in Model 1 with the non-Marketplace sample. For Model 2 with the non-Marketplace sample, the increase in total expenditures (13.8%, *P* = .06), professional expenditures (14.5%, *P* < .01), primary care visits (0.9%, *P* = .12), and specialist visits (3.4%, *P* = .12) were consistent with the estimates for the full sample (see *Appendix Table A3*). A slight difference for Model 2 was that the increases in primary care and specialist visits were no longer statistically significant. Overall, this suggests that the influence of the ACA and the introduction of the Marketplace had minimal effects on the results.

Second, because there may have been a gradual change in costs or utilization as providers adapted to PCMH components, we also explored a comparative interrupted time series approach that uses a linear time trend instead of quarterly fixed effects (see *Appendix Tables A4* *to* *A5*). Using this approach, we found weak evidence in Model 1 of short-term reductions in total, professional, and inpatient facility expenditures and an increase in PCP visits. However, the unadjusted trends are not always linear, which raises concerns about whether there are truly short-term reductions. The linear time trend coefficients that capture longer term change are positive for all study outcomes and statistically significant for total expenditures only (*P* < .1). The positive time trend coefficients are consistent with the main DD models and indicate that any short-term reductions are washed out after a few quarters.

Third, our attribution approach required that a person have at least 3 PCP visits in a given calendar year, and by default, this limited the sample to higher users. This restriction also limited the generalizability of our findings to healthier populations and could introduce measurement error. To assess the potential bias this introduces, we expanded the sample to those who had at least one PCP visit in a given calendar year. The broader sample had lower baseline expenditures and utilization but was similar in demographic and insurance characteristics (see *Appendix Table A6*). We found similar null effects on the study outcomes for the broader sample in Model 1 (see *Appendix Table A7*). For Model 2, we found slightly smaller effects for total and professional expenditures and specialist visits and almost no change in primary care visits. The Model 2 results were consistent with the lower overall expenditures and utilization of the broader sample but still demonstrate an effect on expenditures associated with PCMH adoption.

## Discussion

This study leveraged the recently developed Arkansas APCD to assess spillover effects of the Arkansas Medicaid PCMH Program on the expenditures and service utilization of commercially insured enrollees who received most of their care at a PCMH practice. Arkansas supported permanent, practice-wide transformation for Medicaid PCMH practices that would also change the type of care received by commercial enrollees at Medicaid PCMH practices. We did not find significant spillover effects on expenditures or utilization for the full set of PCMH practices. Among “early adopter” PCMHs certified in early 2014, relative to later adopting practices, we found increases in PCP and specialist visit rates and corresponding increases in total and professional expenditures.

The results for the early adopters are consistent with previously published findings for Arkansas Medicaid beneficiaries attributed to PCMHs in 2014, which showed statistically significant increases in the rate of physician visits.^9^ Our results differ slightly in that the Medicaid findings also show a concomitant decline in inpatient admissions and inpatient and total expenditures. Total expenditures and inpatient admissions rates and their associated expenditures among the commercial population in PCMH practices were also lower than the Medicaid population, which may suggest that there is less of a margin for change among the commercial population. However, Phillips and colleagues note that expenditure reductions may take 3 or more years to materialize.^11^ Three years of follow-up data were only available for early 2014; thus, data from 2017 and beyond would be needed to evaluate long-term change across all cohorts.

Our sensitivity analyses suggest that the results are robust to the influence of the broader implementation of the ACA, but the context of Arkansas and timing during which the PCMH was implemented are still noteworthy for interpreting the results of this study. There was an influx of Marketplace enrollees in 2014, and Marketplace enrollees had substantially higher expenditures and utilization than non-Marketplace enrollees. Overall, this suggests that people with unmet needs were obtaining health care, similar to other large reforms such as the Oregon Health Insurance Experiment.^20^ Given the level of need in the population, the increase in expenditures may in fact be a positive finding given the large increase in access to care.

Programmatic changes in the Medicaid PCMH Program and other reform efforts may affect the long-term sustainability of our results. Arkansas offered practice transformation assistance to participating PCMH practices for up to 24 months after their enrollment date.^19^ Thus, early 2014 adopters would have received “in-kind” transformation support through 2015 but not into 2016 or beyond. More than 70 practices enrolled in the PCMH model worked with a practice transformation coach in 2014, and those that did were more likely to meet the full PCMH requirements during the year. Thus, if the assistance was critical to maintain PCMH requirements, effects in later years may dissipate once the assistance period ends.

Another important change occurred in 2015, when all QHPs participating in the Marketplace were required to provide support for, and align with, the Medicaid PCMH Program or structure their PCMH Program after nationally accepted models. Commercial insurers began paying PMPMs to state-recognized PCMHs for people enrolled in employer-based and self-insured products outside the Marketplace later in 2015, but the exact extent to which PCMHs received payments for their commercially insured patients by the middle of the study period is not readily available. Still, our results for the early adopting practices may suggest that some spillover effects can occur without full commercial payer participation.

There are several limitations for this study. First, we do not have an untreated comparison group due to the aforementioned limitations and rely solely on variation across time. It is possible that some of the null results may reflect this type of design. Second, we did not directly observe the uptake or maintenance of all PCMH components and could not identify heterogeneity in implementation, such as differences between family and pediatric practices. Not all the PCMH requirements were fully implemented in 2014; rather, the requirements were phased in over time. Many practices did not have a sufficient Medicaid panel size to qualify for shared savings—a motivation for increasing quality—and not all practices were continuously enrolled in PCMH.

Third, approximately 20% of practices disenrolled at some point, which affected less than 10% of enrollee quarters. The reasons practices dropped PCMH certification are unknown. Likewise, our attribution approach is annual and contemporaneous, which allows individuals to switch between PCMH practices or drop out of the sample if they are not attributed to a PCMH practice in a given year. If certain types of practices disenroll or if PCMH implementation induces systematic switching of patients, this could bias the results.

Third, we also note that PCMH was introduced in the middle of a long implementation period of state reforms, starting with CPC and most recently with CPC+. Although only 2 practices included this study also participated in CPC during the same period, all study PCMH practices ultimately began participating in the CPC+ initiative in 2017. Therefore, the impacts observed in this study could relate to the broader reforms, including CPC, simultaneously occurring in the state during the same time as the intervention of interest. There may also be some bias associated with the lead-in period up to CPC+ as practices likely made larger, practice-wide changes to prepare for implementing the new multipayer model.

Finally, we note that the APCD does not include self-insured beneficiaries, which could include as much as 65% of the commercially insured market. Given support for statewide transformation efforts from many large self-insured employers, we do not have reason to suspect that the inclusion of individuals with a self-insured plan would affect the results.^21^ However, the extent to which the self-insured population differs from fully insured population may limit the generalizability of our findings.

## Conclusion

PCMHs are increasingly being implemented by policymakers to incentivize high-quality, coordinated care and ultimately lower health care spending. To address several challenges in the health care system, state policymakers in Arkansas implemented permanent delivery reform across multiple payers coinciding with the implementation of the ACA. The state’s Medicaid PCMH Program introduced practice-wide transformation that provided a unique scenario to examine the potential spillover effects on the commercial enrollees. Like results from the Medicaid population, we found increased access to care among commercial beneficiaries but did not find evidence of short-term cost savings or reductions in inpatient hospital admissions. Given the large increase in commercially insured individuals through Medicaid expansion and the Marketplace, it may take more time for savings or reductions in utilization to occur. Even without associated reductions, decisionmakers should not lose sight of the potential value of increased engagement in and coordination of professional services for a population with potentially high unmet health needs. Our results also emphasize that states can leverage Medicaid to spur system-wide transformation, and the investments generate effects beyond those covered directly by Medicaid.

## Supplemental Material

supplemental_material_iq_final – Supplemental material for Did Arkansas’ Medicaid Patient-Centered Medical Home Program Have Spillover Effects on Commercially Insured Enrollees? Click here for additional data file. Supplemental material, supplemental_material_iq_final for Did Arkansas’ Medicaid Patient-Centered Medical Home Program Have Spillover Effects on Commercially Insured Enrollees? by Jesse M. Hinde, Nathan West, Samuel J. Arbes, Marianne Kluckman and Suzanne L. West in INQUIRY: The Journal of Health Care Organization, Provision, and Financing
