# Supplementary material for: Did Arkansas’ Medicaid Patient-Centered Medical Home Program Have Spillover Effects on Commercially Insured Enrollees?
Source: Inquiry. 2020 Jan 16;57:0046958019900753. doi: 10.1177/0046958019900753 (PMC6970475; doi:10.1177/0046958019900753)
Supplement: supplemental_material_iq_final – Supplemental material for Did Arkansas’ Medicaid Patient-Centered Medical Home Program Have Spillover Effects on Commercially Insured Enrollees? [file supplemental_material_iq_final.pdf]

Supplementary Results Appendix

Appendix Table A1

Test of Parallel Assumptions for Utilization and Expenditures in 2013: Arkansas Commercial Enrollees Not Enrolled through the Marketplace, 2013–2016

Study Outcome	Weighted N	Early 2014 PCMH change in slope estimate (SE)	Mid-2014 PCMH change in slope estimate (SE)	2015 PCMH change in slope estimate (SE)
Expenditures				
Total expenditures	121,516	-0.2	-1.4	97.3***
		(9.638)	(12.178)	(35.384)
Professional expenditures	121,516	0.9	4.3	9.2*
		(3.625)	(3.452)	(4.997)
Pharmaceutical expenditures	121,516	-1.1	-1.5	6.1
		(1.880)	(2.021)	(6.557)
Inpatient facility expenditures	121,516	2.1	4.9	76.8**
		(4.800)	(7.095)	(37.359)
Outpatient facility expenditures	121,516	-2.1	-9.0	5.2
		(3.717)	(8.927)	(6.302)
Service utilization				
Inpatient admissions per 1,000 enrollee quarters	115,597	-0.3	0.5	2.7**
		(0.001)	(0.001)	(0.001)
Primary care visits per 100 enrollee quarters	121,516	0.6	0.8	0.5
		(0.005)	(0.006)	(0.008)
Specialist visits per 100 enrollee quarters	121,488	0.1	-0.2	0.3
		(0.003)	(0.003)	(0.004)
Emergency department visits per 1,000 enrollee quarters	121,404	-1.9	0.4	-3.1
		(0.002)	(0.002)	(0.002)

PCMH = patient-centered medical home.

Notes: Comparative interrupted time series regression models were estimated using ordinary least squares for the expenditure outcomes and maximum likelihood logit for the service use outcomes. The change in slope estimates represents the difference in the linear trend relative to the 2016 PCMH group. Standard statistical practice is to use confidence intervals of 90% or higher.

Source: Arkansas All-Payer Claims Database, 2013.

Appendix Table A2

Weighted Annual Sample Characteristics by Marketplace Status, 2013–2016

Characteristic	Non-Marketplace population annualized by PCMH status		Non-Marketplace population annualized by calendar year				Marketplace-only population annualized by calendar year		
	Pre-PCMH [1]	Post-PCMH [2]	2013 [3]	2014 [4]	2015 [5]	2016 [6]	2014 [7]	2015 [8]	2016 [9]
Total expenditures	$3,353	$3,881	$3,427	$3,612	$3,904	$4,328	$7,330	$8,523	$8,944
Professional expenditures	$1,349	$1,483	$1,400	$1,459	$1,487	$1,575	$2,732	$2,868	$2,883
Prescription expenditures	$805	$1,046	$835	$880	$1,064	$1,204	$1,230	$1,591	$1,746
Inpatient facility expenditures	$466	$488	$459	$448	$503	$576	$1,617	$1,917	$2,033
Other facility expenditures	$734	$864	$734	$826	$849	$973	$1,752	$2,147	$2,282
Any inpatient admission, %	3.0	3.4	3.1	3.0	3.5	3.9	10.7	12.8	12.6
Any specialist visit, %	42.3	46.3	43.0	44.6	46.9	48.4	49.3	52.1	53.3
Any ED visit, %	20.4	22.0	20.7	21.4	22.6	23.4	43.9	45.1	44.4
Age	29.6	30.9	28.0	29.7	31.5	32.3	38.9	39.3	39.8
Female, %	55.5	55.6	55.2	55.7	55.5	55.9	70.4	67.5	67.4
BH diagnosis, %	17.9	18.3	18.5	18.4	18.8	18.4	29.9	31.5	32.9
Lives in MSA, %	55.3	57.1	57.2	53.6	58.0	56.7	48.4	44.6	42.7
Has prescription drug coverage, %	83.8	82.6	83.9	83.7	81.5	82.8	100.0	100.0	100.0
Insurance product type									
Other commercial insurance, %	14.3	11.2	14.2	14.5	9.8	10.2	20.9	30.3	34.7
PPO, %	48.3	51.7	49.8	51.3	51.8	50.3	79.1	69.7	65.3
PoS, %	27.5	25.1	27.2	25.1	25.8	25.0	0.0	0.0	0.0
Insurance market type									
Individual market plan, %	28.9	30.7	27.8	28.6	31.8	31.8	100.0	100.0	100.0
Large employer plan, %	42.1	42.9	42.7	41.8	43.0	43.4	0.0	0.0	0.0
Small employer plan, %	11.6	11.7	11.8	11.7	11.8	11.3	0.0	0.0	0.0
Unweighted N	42,732	77,567	30,989	30,176	28,272	27,453	15,945	23,484	25,047
Weighted N	44,261	80,102	32,107	31,102	29,121	28,480	17,744	24,957	26,548

BH = behavioral health; ED = emergency department; MSA = metropolitan statistical area; PCMH = patient-centered medical home; PPO = preferred provider organization; PoS = point of service.

Source: Arkansas All-Payer Claims Database, 2013–2016.

Appendix Table A3

Estimated Spillover Effects of Medicaid PCMH Adoption on PMPM Expenditures and Quarterly Rates of Utilization ***for the non-Marketplace sample****, 2013–2016*

		Model 1: Spillover effects across all PCMH practices			Model 2: Spillover effects for early 2014 PCMH practices		
**Study outcome**	**Weighted N**	**2013 Adjusted mean**	**DD estimate (SE)**	**Relative difference (%)**	**2013 Adjusted mean, CG practice**	**DD estimate (SE)**	**Relative difference (%)**
Expenditures							
Total expenditures	458,622	292.8	6.5	2.2	344.8	37.9*	13.8
			(12.213)			(20.195)	
Professional expenditures	458,622	119.4	2.4	2.0	133.0	16.6***	14.5
			(3.518)			(5.179)	
Pharmaceutical expenditures	458,622	71.1	3.0	4.2	77.6	2.9	4.2
			(3.699)			(7.829)	
Inpatient facility expenditures	458,622	39.9	-1.2	-3.0	58.9	13.7	41.0
			(6.906)			(14.438)	
Outpatient facility expenditures	458,622	62.4	2.3	3.7	75.3	4.7	8.1
			(5.409)			(7.184)	
Service utilization							
Inpatient admissions per 1,000 enrollee quarters	453,099	9.1	0.6	6.2	11.7	0.0	0.6
			(0.001)			(0.001)	
Primary care visits per 100 enrollee quarters	458,622	70.5	0.2	0.3	71.8	0.7	0.9
			(0.004)			(0.004)	
Specialist visits per 100 enrollee quarters	458,594	18.6	-0.2	-1.1	19.6	0.6	3.4
			(0.004)			(0.004)	
Emergency department visits per 1,000 enrollee quarters	458,575	67.0	1.8	2.7	71.6	1.8	2.7
			(0.002)			(0.003)	

DD = difference-in-differences; PCMH = patient-centered medical home; PMPM = per member per month; SE= standard error; CG = comparison group.
* p<0.1, ** p<0.05, *** p<0.01.

Notes: DD models were estimated using ordinary least squares for expenditure outcomes and maximum likelihood logits for service utilization outcomes. The relative difference is the DD estimate expressed as a percentage of the 2013 adjusted mean.
Source: Arkansas All-Payer Claims Database, 2013–2016

Appendix Table A4

Estimated Spillover Effects of Medicaid PCMH Adoption on PMPM Expenditures and Quarterly Rates of Utilization ***using comparative interrupted time series for Model 1 (Effect across all PCMH practices)****, 2013–2016*

Study outcome	Weighted N	2013 Adjusted mean	DD estimate (SE)	Relative difference (%)	Change in slope estimate (SE)
Expenditures					
Total expenditures	704,419	289.2	-49.5**	-17.1	6.0*
			(21.028)		(3.4476)
Professional expenditures	704,419	120.1	-1.1	-0.9	0.1
			(5.503)		(0.8621)
Pharmaceutical expenditures	704,419	70.0	-7.7	-11.0	1.6
			(7.183)		(1.1031)
Inpatient facility expenditures	704,419	38.2	-25.9**	-67.7	2.4
			(10.124)		(1.7548)
Outpatient facility expenditures	704,419	61.0	-14.8	-24.3	1.9
			(9.003)		(1.5581)
Service utilization					
Inpatient admissions per 1,000 enrollee quarters	701,897	8.6	0.3	3.3	0.1
			(0.001)		(0.0001)
Primary care visits per 100 enrollee quarters	704,416	70.5	0.9*	1.3	0.1
			(0.005)		(0.0007)
Specialist visits per 100 enrollee quarters	704,384	18.4	-0.2	-0.9	0.1
			(0.005)		(0.0009)
Emergency department visits per 1,000 enrollee quarters	704,373	66.4	3.3	5.0	-0.2
			(0.002)		(0.0004)

DD = difference-in-differences; PCMH = patient-centered medical home; PMPM = per member per month; SE= standard error.
* p<0.1, ** p<0.05, *** p<0.01.
Notes: A comparative interrupted time series model was estimated using ordinary least squares for expenditure outcomes and maximum likelihood logits for service utilization outcomes. The relative difference is the DD estimate expressed as a percentage of the 2013 adjusted mean.

Source: Arkansas All-Payer Claims Database, 2013–2016.

Appendix Table A5

Estimated Spillover Effects of Medicaid PCMH Adoption on PMPM Expenditures and Quarterly Rates of Utilization ***using comparative interrupted time series for Model 2 (Effect for early 2014 practices)****, 2013–2016*

Study outcome	Weighted N	2013 Adjusted mean, CG practices	DD estimate (SE)	Relative difference (%)	Change in slope estimate (SE)
Expenditures					
Total expenditures	704,419	325.9	-3.0	-1.1	7.3
			(41.480)		(5.418)
Professional expenditures	704,419	131.9	8.7	7.6	0.3
			(7.518)		(1.847)
Pharmaceutical expenditures	704,419	73.4	-20.9	-30.4	5.6***
			(16.691)		(2.032)
Inpatient facility expenditures	704,419	50.5	-0.1	-0.1	2.3
			(24.496)		(3.699)
Outpatient facility expenditures	704,419	70.0	9.2	15.9	-1.0
			(9.401)		(2.287)
Service utilization					
Inpatient admissions per 1,000 enrollee quarters	701,897	9.8	7.0***	85.6	1.3***
			(0.002)		(0.000)
Primary care visits per 100 enrollee quarters	704,416	71.4	7.1***	10.1	1.3***
			(0.014)		(0.002)
Specialist visits per 100 enrollee quarters	704,384	18.9	-0.7	-3.6	-0.1
			(0.012)		(0.002)
Emergency department visits per 1,000 enrollee quarters	704,373	68.9	10.7	16.4	2.4*
			(0.009)		(0.001)

DD = difference-in-differences; PCMH = patient-centered medical home; PMPM = per member per month; SE= standard error.
* p<0.1, ** p<0.05, *** p<0.01.
Notes: A comparative interrupted time series model was estimated using ordinary least squares for expenditure outcomes and maximum likelihood logits for service utilization outcomes. The relative difference is the early 20140 DD estimate expressed as a percentage of the 2013 adjusted comparison group mean.

Source: Arkansas All-Payer Claims Database, 2013–2016.

Appendix Table A6

*Weighted Annual Sample Characteristics for the **sample attributed with a one-visit floor**, 2013–2016*

Sample characteristic	Annualized by PCMH status		Annualized by calendar year				Annualized by PCMH group in the baseline period (2013)			
	Pre-PCMH [1]	Post-PCMH [2]	2013 [3]	2014 [4]	2015 [5]	2016 [6]	Early 2014 PCMH adopters [7]	Mid-2014 PCMH adopters [8]	2015 PCMH adopters [9]	2016 PCMH adopters [10]
Total expenditures	$2,408	$3,647	$1,982	$3,051	$3,833	$4,227	$1,891	$2,488	$2,483	$3,459
Professional expenditures	$948	$1,303	$829	$1,197	$1,359	$1,433	$803	$952	$946	$1,369
Prescription expenditures	$533	$794	$498	$622	$829	$930	$481	$589	$639	$698
Inpatient facility expenditures	$397	$693	$246	$538	$747	$846	$222	$379	$410	$576
Other facility expenditures	$531	$857	$410	$693	$898	$1,018	$384	$568	$489	$816
Any inpatient admission, %	2.9	5.0	2.0	3.9	5.5	5.8	1.8	2.7	2.7	4.6
Any specialist visit, %	27.6	32.8	27.3	30.7	33.5	34.8	27.1	32.8	25.5	28.1
Any ED visit, %	17.1	23.4	14.2	21.6	24.7	25.4	14.0	14.4	16.8	26.0
Age	30.1	32.5	27.1	31.1	33.2	33.8	25.8	32.4	33.2	35.9
Female, %	53.7	56.1	51.6	55.8	56.1	56.7	51.3	52.9	55.7	60.7
BH diagnosis, %	13.2	16.4	12.1	15.2	17.0	17.8	12.0	10.9	14.2	18.9
Lives in MSA, %	53.3	53.1	56.8	52.5	52.8	50.9	59.1	56.7	34.2	33.0
Has prescription drug coverage, %	86.7	88.9	84.2	88.8	88.6	89.8	83.6	86.5	90.0	94.4
Marketplace plan, %	15.9	19.0	14.5	17.0	18.7	21.4	13.4	15.5	16.2	16.1
Insurance product type										
Other commercial insurance, %	15.9	19.0	14.5	17.0	18.7	21.4	13.4	15.5	16.2	16.1
Insurance type—PPO, %	54.9	58.8	51.9	60.3	59.2	57.0	54.0	45.5	48.2	63.5
Insurance type—PoS, %	23.2	16.2	27.3	17.9	16.1	14.7	26.5	31.1	27.7	15.1
Insurance market type										
Individual market plan, %	40.1	55.4	28.0	49.3	58.1	59.8	27.3	33.8	31.4	60.5
Large employer plan, %	35.2	27.3	42.4	29.4	26.2	25.2	43.3	39.1	40.6	23.1
Small employer plan, %	9.8	7.6	11.7	8.2	7.4	6.7	11.7	12.3	12.4	7.1
Unweighted N	116,256	270,034	72,434	97,178	102,940	101,921	54,425	7,324	2,984	5,444
Weighted N	123,057	284,626	75,497	103,082	108,349	107,681	56,737	7,612	3,126	5,857

BH = behavioral health; ED = emergency department; MSA = metropolitan statistical area; PCMH = patient-centered medical home; PoS = point of service; PPO = preferred provider organization.

Notes: Numbers in brackets refer to the column numbers. Column numbers are referenced in the text discussing this exhibit.

Source: Arkansas All-Payer Claims Database, 2013.

Appendix Table A7

Estimated Spillover Effects of Medicaid PCMH Adoption on PMPM Expenditures and Quarterly Rates of Utilization ***using a sample attributed with a one-visit floor****, 2013–2016*

		Model 1: Spillover effects across all PCMH practices			Model 2: Spillover effects for early 2014 PCMH practices		
**Study outcome**	**Weighted N**	**2013 Adjusted mean**	**DD estimate (SE)**	**Relative difference (%)**	**2013 Adjusted mean, CG practice**	**DD estimate (SE)**	**Relative difference (%)**
Expenditures							
Total expenditures	1,440,629	171.4	-0.1	-0.1	196.7	1.3	0.8
			(7.734)			(15.055)	
Professional expenditures	1,440,629	71.7	-1.3	-1.9	78.4	6.5*	9.3
			(2.389)			(3.776)	
Pharmaceutical expenditures	1,440,629	42.9	3.2	7.4	47.3	-1.6	-3.8
			(2.313)			(4.321)	
Inpatient facility expenditures	1,440,629	21.5	-2.9	-13.3	29.7	2.5	13.4
			(4.709)			(6.887)	
Outpatient facility expenditures	1,440,629	35.3	0.9	2.6	41.3	-6.2	-18.5
			(3.643)			(6.335)	
Service utilization							
Inpatient admissions per 1,000 enrollee quarters	1,440,629	5.6	0.1	1.6	6.8	-0.4	-7.0
			(0.0005)			(0.001)	
Primary care visits per 100 enrollee quarters	1,440,629	50.5	0.3	0.6	51.4	0.1	0.1
			(0.003)			(0.007)	
Specialist visits per 100 enrollee quarters	1,440,629	10.7	-0.3	-2.8	11.2	0.0	-0.3
			(0.003)			(0.004)	
Emergency department visits per 1,000 enrollee quarters	1,440,629	44.3	1.2	2.7	47.1	2.2	5.1
			(0.002)			(0.003)	

DD = difference-in-differences; PCMH = patient-centered medical home; PMPM = per member per month; SE= standard error; CG = comparison group.
* p<0.1, ** p<0.05, *** p<0.01.

Notes: DD models were estimated using ordinary least squares for expenditure outcomes and maximum likelihood logits for service utilization outcomes. The relative difference is the DD estimate expressed as a percentage of the 2013 adjusted mean.
Source: Arkansas All-Payer Claims Database, 2013–2016

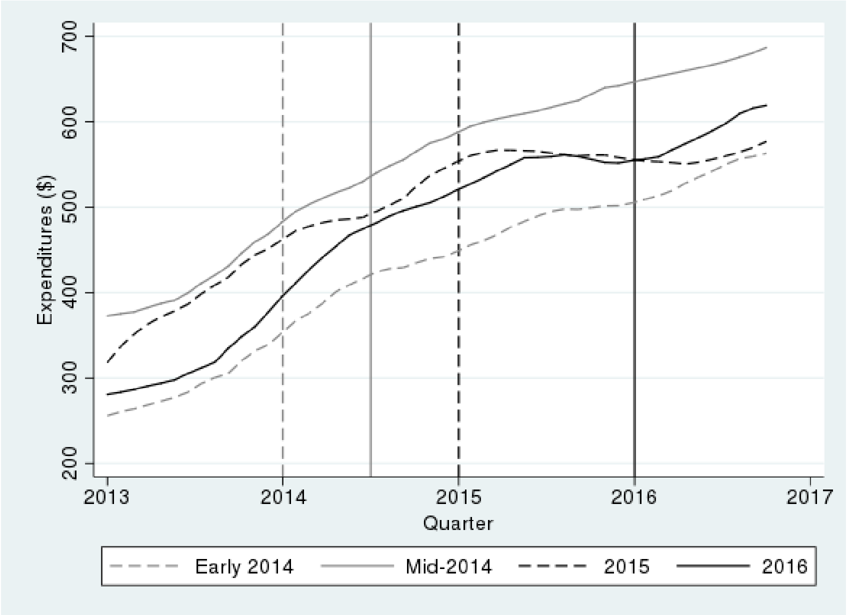

Appendix Figure A1. Unadjusted quarterly total expenditures by PCMH group (2013–2016). Source: Arkansas All-Payer Claims Database, 2013–2016.
PCMH = patient-centered medical home.
Note: Quarterly trends were smoothed using local polynomial regression with an Epanechnikov kernel.

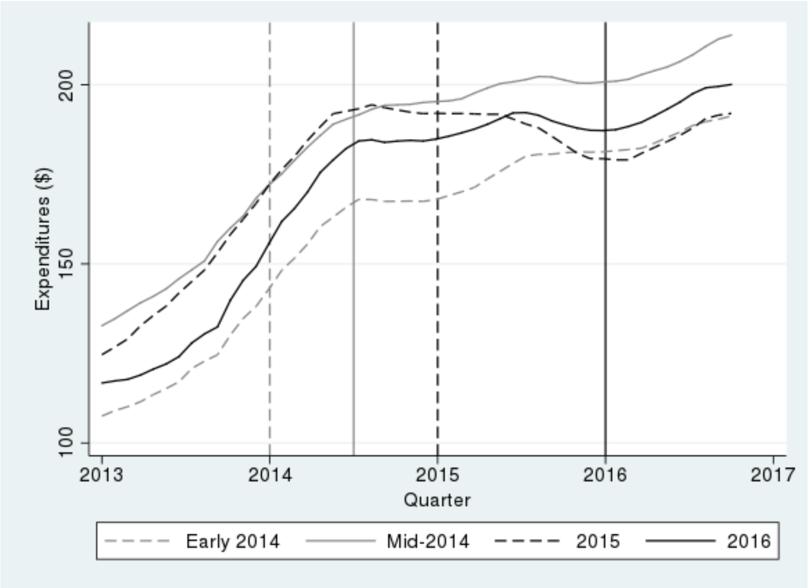

Appendix Figure A2. Unadjusted quarterly professional expenditures by PCMH group (2013–2016). Source: Arkansas All-Payer Claims Database, 2013–2016.
PCMH = patient-centered medical home.
Note: Quarterly trends were smoothed using local polynomial regression with an Epanechnikov kernel.

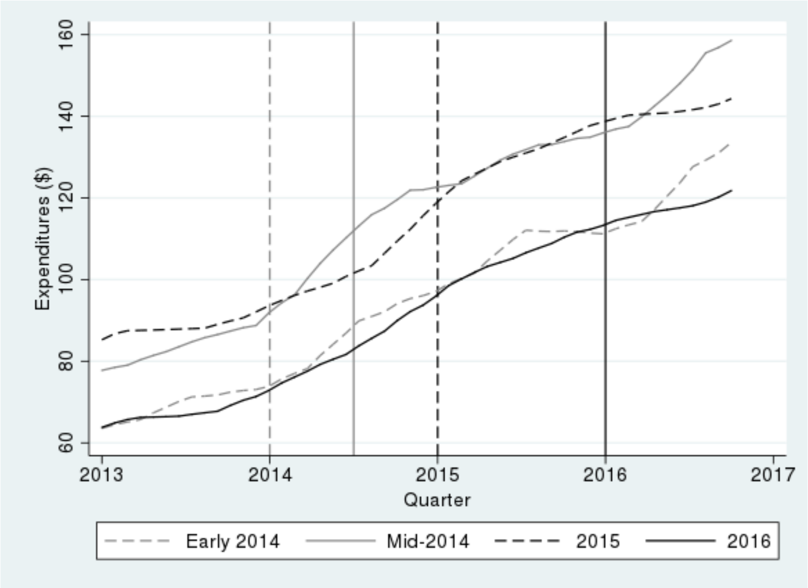

Appendix Figure A3. Unadjusted quarterly prescription drug expenditures by PCMH group (2013–2016). Source: Arkansas All-Payer Claims Database, 2013–2016.
PCMH = patient-centered medical home.
Note: Quarterly trends were smoothed using local polynomial regression with an Epanechnikov kernel.

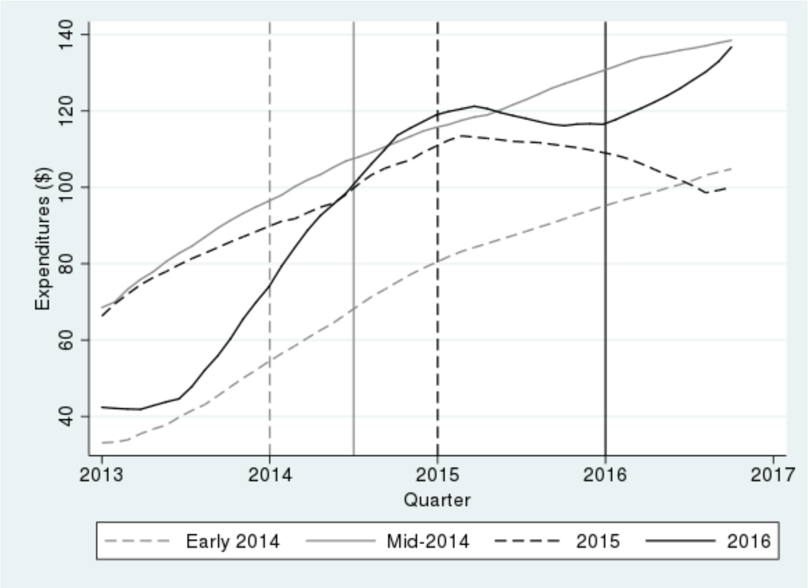

Appendix Figure A4. Unadjusted quarterly inpatient facility expenditures by PCMH group (2013–2016). Source: Arkansas All-Payer Claims Database, 2013–2016.
PCMH = patient-centered medical home.
Note: Quarterly trends were smoothed using local polynomial regression with an Epanechnikov kernel.

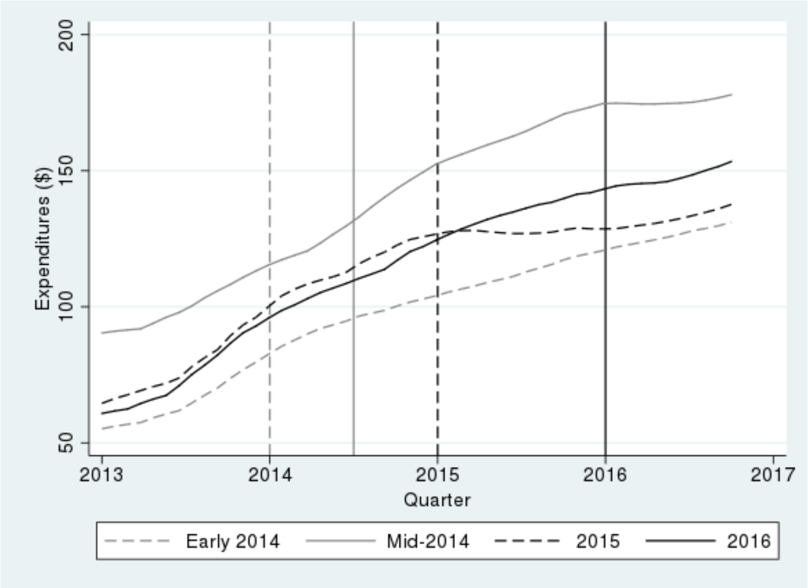

Appendix Figure A5. Unadjusted quarterly non-inpatient facility expenditures by PCMH group (2013–2016). Source: Arkansas All-Payer Claims Database, 2013–2016.
PCMH = patient-centered medical home.
Note: Quarterly trends were smoothed using local polynomial regression with an Epanechnikov kernel.

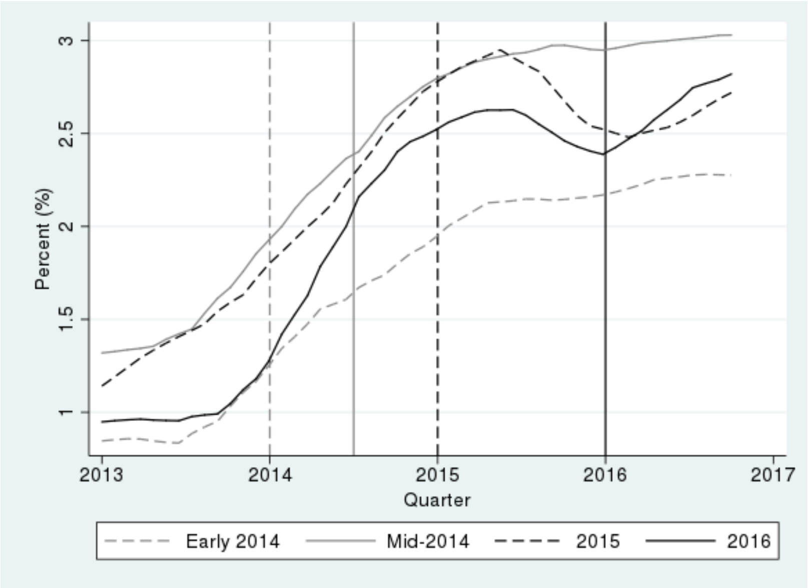

Appendix Figure A6. Unadjusted quarterly proportion with an inpatient admission, by PCMH group (2013–2016). Source: Arkansas All-Payer Claims Database, 2013–2016.
PCMH = patient-centered medical home.
Note: Quarterly trends were smoothed using local polynomial regression with an Epanechnikov kernel.

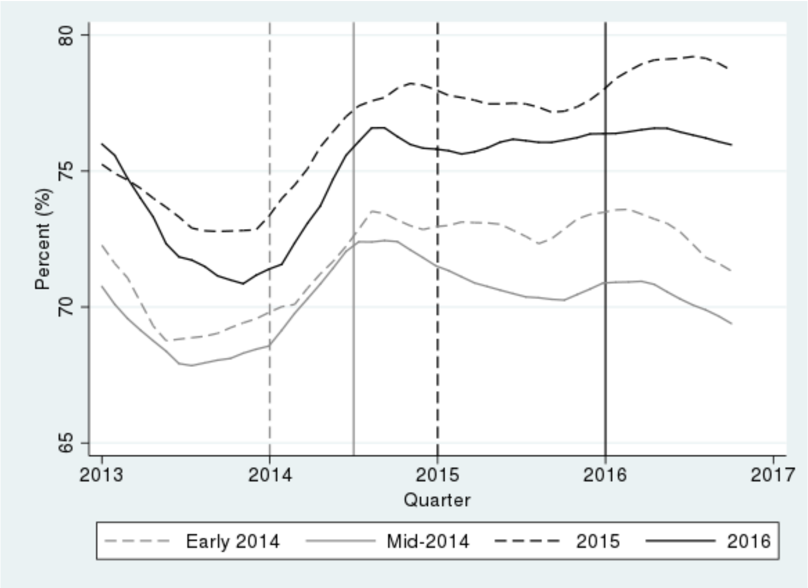

Appendix Figure A7. Unadjusted quarterly proportion with a primary care visit, by PCMH group (2013–2016). Source: Arkansas All-Payer Claims Database, 2013–2016.
PCMH = patient-centered medical home.
Note: Quarterly trends were smoothed using local polynomial regression with an Epanechnikov kernel.

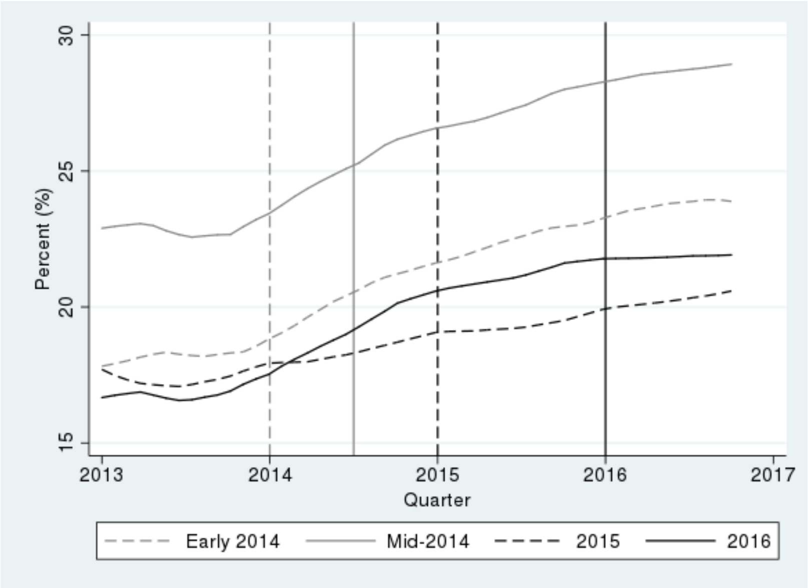

Appendix Figure A8. Unadjusted quarterly proportion with a specialist visit, by PCMH group (2013–2016). Source: Arkansas All-Payer Claims Database, 2013–2016.
PCMH = patient-centered medical home.
Note: Quarterly trends were smoothed using local polynomial regression with an Epanechnikov kernel.

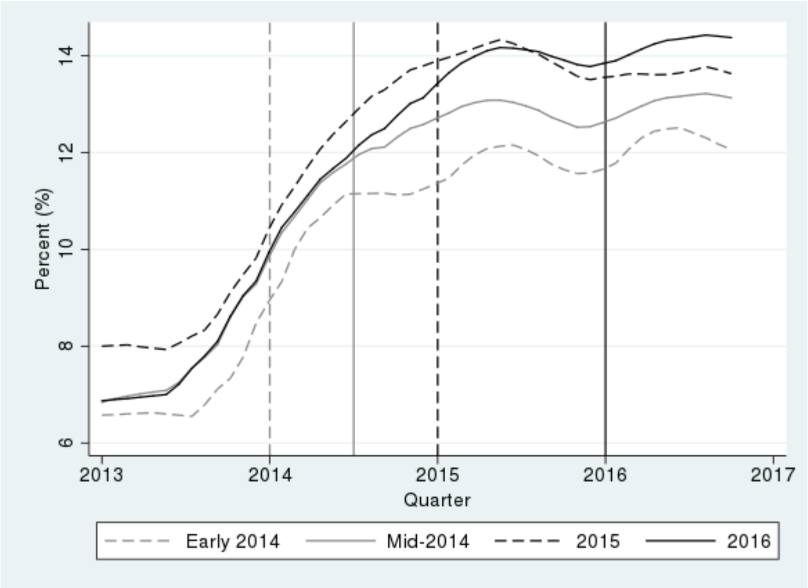

Appendix Figure A9. Unadjusted quarterly proportion with an emergency department visit, by PCMH group (2013–2016). Source: Arkansas All-Payer Claims Database, 2013–2016.
PCMH = patient-centered medical home.
Note: Quarterly trends were smoothed using local polynomial regression with an Epanechnikov kernel.
